# “It’s what mothers do.” A qualitative exploration of mothers’ experiences of supporting their daughter to be physically active

**DOI:** 10.1371/journal.pone.0299260

**Published:** 2024-04-01

**Authors:** Carol Brennan, Grainne O’Donoghue, Alison Keogh, Ryan E. Rhodes, James Matthews

**Affiliations:** 1 School of Public Health, Physiotherapy, and Sports Science, University College Dublin, Dublin, Ireland; 2 School of Medicine, Trinity College Dublin, The University of Dublin, Dublin, Ireland; 3 School of Exercise Science, Physical and Health Education, University of Victoria, Victoria, Canada; 4 Institute of Sport and Health, University College Dublin, Dublin, Ireland; University of Greenwich, UNITED KINGDOM

## Abstract

**Objective:**

Girls are more at risk than boys of the non-communicable diseases associated with insufficient levels of physical activity (PA), therefore it is important to explore the reasons why girls maintain or cease to be physically active. Maternal support plays an important role in girl’s PA, yet the factors influencing mothers’ support of their PA have received limited exploration. In response, the aim of this study was to explore, mothers’ experiences of supporting their daughters to be physically active and their perceptions of the factors that might influence these experiences.

**Method:**

Semi-structured interviews were conducted with a purposive sample of mothers (n = 29) of girls (Mean age = 10.9 years; SD = 0.6). Reflexive Thematic Analysis was used to analyse the data, with themes mapped to the relevant domains of the Theoretical Domains Framework.

**Results:**

Themes highlighted how mothers described providing PA support as an inherent part of their parental role and how their role was influenced by their own PA identity. Mothers recognised that the type and amount of support they provided was impacted by the community setting in which they lived. Mothers acknowledged how the role of others (e.g., partners, grandparents, peers) added a layer of complexity to supporting their daughters to be active.

**Conclusion:**

This study advances our understanding of maternal PA support behaviours recognising the complex interplay of individual, social and environmental factors. Additionally, the use of the Theoretical Domains Framework presents an in-depth behavioural diagnosis which can be used to inform future theory-based interventions to promote parent support of children’s PA.

## 1. Introduction

Regular physical activity (PA) provides a range of health benefits to children and adolescents [1]. However, despite these benefits, PA levels are low and typically regress annually throughout adolescence [2, 3]. This rate of decline is most apparent during the transition period from primary to secondary school [4, 5], with no more than one third of children and adolescents meeting the World Health Organisation’s recommendation to engage in at least an average of 60 minutes of moderate to vigorous-intensity, mostly aerobic, physical activity, per day [6]. This gap is more pronounced in girls than boys [4, 7]. For example, in Latin America, boys are twice as likely to meet PA guidelines as girls [8] whereas in Ireland, 11% of girls meet PA guidelines as compared to 19% of boys [9]. Consequently, girls are more at risk of the many non-communicable diseases associated with insufficient levels of PA [10], such as obesity, type II diabetes and cardiovascular disease [11, 12]. Therefore, it is important to explore the reasons why girls maintain or cease to be physically active [13] during this seminal phase of life with a view to promoting lifelong PA participation [14].

Children’s PA is a complex behaviour influenced by multiple individual (e.g., age, gender, motivation), social (e.g., friend support, family support) and environmental factors (e.g., facilities, cost) [15–17]. One established factor is the essential role parents play in the development of children’s PA [18, 19]. Parental support is an umbrella term used to represent numerous support behaviours for PA such as encouragement (e.g., offering praise, feedback and spectating), logistical support (e.g., provision of transport), co-activity (e.g., parents providing support through being active with their child), environmental support (e.g., provision of materials and equipment necessary to facilitate PA), and regulatory support (e.g., establishing rules and regulations around PA) [20, 21].

There is a body of research showing that parental support is positively related to child and adolescent PA [22–24].This is particularly evident for children aged between 10–12 years (i.e., tweens) as this is a stage where parents perceive they can still shape their children’s activity in positive ways [25, 26]. Typically, research suggests mothers engage in varying PA support behaviours more frequently than fathers [26, 27], and that children with active mothers are more likely to be active themselves [28]. This relationship between mothers engagement in supportive behaviours and children’s level of PA appears to be more pronounced between mother-daughters dyads as compared to mother-son dyads or father-daughter dyads [22, 23], highlighting the important role of mothers in promoting PA to girls. While these studies contribute to our knowledge of maternal PA support behaviours, there are gaps in our understanding of the factors influencing these behaviours [20], for example, most of these studies have been quantitative in nature which has limitations in the depth of understanding it provides [29]. Thus, there is a need for further qualitative research that includes mothers’ own voices and how they negotiate supporting their daughters to be physically active [30].

While there has been limited qualitative research exploring mothers’ support behaviours, an extensive body of qualitative research has explored mothers’ experiences of engaging in PA [31–35]. Many of these studies adopt a social constructivist approach with motherhood “viewed as the product of individual, social and cultural discourses which interact to create particular meanings concerning mother identity” (McGannon et al. 2018: 41) [34]. This identity is underpinned by the “ethic of care” concept whereby women focus on satisfying the needs of their children first [31] with many mothers only likely to participate in PA when it is formally constructed as being part of a “good mother” [33]. This identity as a “good mother” is based upon the gender stereotypes and gender roles that exist in society. Thus, women’s PA levels and their related PA identity may be negatively influenced by societal expectations and cultural norms to engage in certain roles [36]. Indeed, a recent review examining the barriers and facilitators to PA in women highlighted that women typically hold multiple roles (e.g., employee, partner, and parent), and experience pressure from society to fulfil these roles. Women are also discouraged from seeking fulfilment outside of these traditional roles through the formation of alternative identities such as being an exerciser or active person [37]. Consequently, the development or maintenance of a PA identity may prove challenging because as women age and transition through life stages (e.g., transition to employment, to committed relationships, to motherhood), there is an expectation to engage in multiple traditional roles which often overlap and can limit their ability to develop alternative identities [37, 38]. Thus, a women’s PA identity is only relative to the strength of her other relevant identities (e.g., being a mother or caregiver) and which form her overall sense of self [39, 40]. Importantly, mothers’ PA identity is not only relevant for their own behaviour but also for their children, as family is the primary source from which children draw their gender related stereotypes, learning the social rules that suggest what girls and boys must do [41]. These gender stereotypes effects on children may be reduced when certain strategies are applied, e.g., the availability of positive role models [42]. Therefore, deepening our understanding of mothers’ engagement in support behaviours such as encouragement, co-activity, environmental, and regulatory support through qualitative research might suggest how we can reduce these stereotype effects, and further foster PA in girls.

In exploring mothers’ support behaviours, there is increasing recognition of the need for theoretical perspectives that look beyond examining the individual in isolation and considers the social, cultural, and environmental contexts which can influence behaviour [33]. Indeed, researchers have underlined the importance of understanding the sources and settings linking family and PA behaviours of children and adolescents within a broad socioecological context [43, 44]. One framework which might provide a useful lens through which to explore mothers’ support behaviours is the Theoretical Domains Framework (TDF) [45]. The TDF contains 14 domains examining psychological (e.g., beliefs about capabilities, identity), social (e.g., social influences), and environmental (e.g., environmental context) influences on behaviour and has been utilised in qualitative research exploring children and adolescents’ physical activity. For example, the TDF has informed studies examining the barriers and facilitators to physical activity as perceived by children, parents and physical education teachers [46, 47] and it has also been used to inform intervention development studies such as the design of a school-based intervention targeting adolescent girls [48]. This framework does not propose testable relationships between factors but provides a lens through which to view different influences on behaviour [49], thus, guiding analysis rather than imposing a predetermined structure [50].

Parental support is positively related to children’s PA, with mothers engaging more frequently in providing PA support than fathers, particularly to girls. However, there is a lack of clarity as to the factors that influence mothers’ engagement in these supportive behaviours, and limited exploration of mothers’ experiences of providing this support to their daughters using qualitative means. Furthermore, there has been a lack of theory applied to examining maternal support behaviours that looks beyond the intra-individual factors to consider how social, cultural and environment factors might influence these behaviours. Therefore, the purpose of the current study was to explore, mothers’ experiences of supporting their daughters to be physically active, and their perceptions of the factors that might influence these experiences, using the TDF as a theoretical lens.

## 2. Method

### 2.1 Research design and philosophical approach

The aim of this study was to explore the phenomenon of mothers supporting their daughters to be physically active and to identify the factors that might influence their experiences. In order to uncover the subjective reality of participants, qualitative methods were selected, with semi-structured interviews as the method of data collection [51]. Interviews were chosen as the data collection method due to their flexibility in allowing participants to expand on the meanings they attribute to their experiences, enabling the interviewer to gain a more thorough understanding [52]. In utilising this research design, a critical realist position was adopted. Critical realism espouses that a reality exists independent of our construction of it, while maintaining that our knowledge of reality is interpretive, partial and fallible [53]. Specifically, critical realism views reality as divided into three levels, the empirical level which is what can be observed or experienced, the actual level where events occur whether we experience them or not and the real level, where the structures and causal mechanisms within an object that lead to the production of events exist [54]. Critical realism attempts to explain these phenomena or social events (i.e., mothers’ experiences of providing PA support) by understanding the causal mechanisms that contribute to these events [55].

A critical realist stance is coherent with the use of qualitative research by guiding researchers to concentrate on describing the prevailing social relations that generate real-world problems, providing the opportunity to produce impactful research [56]. For example, adopting a critical realist position can help us to understand why and how mothers might engage in PA support behaviours with their pre-teen daughters and how this knowledge might contribute to the development of an intervention targeting an increase in pre-teen girls PA. The role of theory in critical realism is well established with an emphasis on the importance of theory for rigorous inquiry [57]. The use of theory enables researchers to articulate relevant causal mechanisms, providing explanations for why events occur as they do [54]. Whilst recognising the role of theory in critical realism, the use of theoretical frameworks should remain flexible enough to accommodate different perspectives that may contribute to a more nuanced understanding of causality. Thus, the TDF was used to inform the study both in the data collection and data analysis phases due to its it relative broadness and non-prescribed directionality between domains [51]. Taking a critical realist stance, reflexive thematic analysis was chosen as the method of data analysis as it would enable us to interpret mother’s experiences of supporting their daughter to be physically active, the context of these experiences, and unpack the key factors that contributed to these experiences [51]. Finally, as reflexive researchers we recognised that our autobiographies and values would shape the study design, information shared in the interviews, the analytic process and the reporting of the data [50]. It is worth noting that no member of the research team was a parent of a girl aged 10–12 years, however our research team was created based on areas of expertise, research interest and experiences, and collectively we brought a range of perspectives to this topic.

### 2.2 Participants and recruitment

A purposive sampling approach was employed to identify mothers of girls based on the following criteria (1) their daughter/s was aged between 10–12 years and (2) daughter/s attended a primary school in the Leinster province of Ireland. Sampling aimed to be representative of both urban and rural regions and to recruit both physically active and inactive mothers. Participants included 29 mothers of girls, aged 10–12 years. See Table 1 for a summary of demographic characteristics of the sample.

**Table 1:** Demographic information of the 29 mothers participating in the interviews.

Demographics		n (%)
**Nationality**	Irish	27 (93)
	Other	2 (7)
**Highest education level**	Non-completion of secondary school	1 (3)
	Completion of secondary school	7 (24)
	Post-secondary school certificate	4 (14)
	University / Institute of Technology degree / diploma	17 (59)
**Employment status**	Not currently employed	4 (14)
	Part-time	7 (24)
	Full-time	18 (62)
**Home location**	Rural	12 (41)
	Urban	17 (59)
**Physical activity level** *	Insufficiently active	11 (38)
	Moderately active	5 (17)
	Active	13 (45)
**Daughter’s participation in organised sport/ after-school activities**	Yes	22 (71)
	No	9 (29)
**Age (years)**	Mean (Range; SD)	42.6 (34–52; 4.0)
**Number of children**	Mean (Range; SD)	2.4 (1–4; 0.59)

Note. * As measured by Godin’s Leisure Time Exercise Questionnaire

### 2.3 Procedures

The Standards for Reporting Qualitative Research (SRQR) guidelines were used to report this study [58] (see S1 File). Ethical approval was obtained by a University Human Research Ethics Committee (LS-21-45) prior to study commencement. An initial email describing the study was distributed to the schools. School principals then invited mothers to take part in the study and an interview date and time was arranged with prospective participants. After providing written informed consent, mothers were invited to complete a demographic questionnaire and participate in an interview. Interviews were conducted via conferencing software due to Covid-19 pandemic social distancing restrictions [59]. To generate rich interactions, CB spent time in conversations prior to each interview with the mothers to establish rapport [60] and provided a lay definition of PA for clarity and understanding. All interviews were recorded and transcribed verbatim. Sample size was determined using the principles of information power, where study aims, sample specificity, use of theory, quality of dialogue and analysis strategy are considered [61]. Ongoing dialogue by members of the research team during data collection and analysis ensured a suitable sample size had been obtained to achieve the study aims.

### 2.4 Interview schedule

A semi-structured interview guide, with three sections was developed by CB and JM. Section A, contained introductory questions to find out mothers’ perspectives on PA and how active their daughters are. Section B focused on how mothers support their daughters to be active and the factors influencing this support, and Section C ended with summary questions and invited the mothers to add anything else they felt was relevant. To respect conversation flow and facilitate a comprehensive understanding of the factors related to maternal PA support, broad open-ended questions based on the 14 TDF domains were used [62] (see S2 File). Three pilot interviews were conducted to trial the interview schedule. As a result of piloting, some interview questions were rephrased, and additional probing questions were added to the guide to encourage mothers to elaborate further on their experiences. All interviews were conducted by the first author. Interviews took place between September and December 2021, mean duration was 32 minutes (SD = 8 minutes).

### 2.5 Data analysis

Microsoft Word^TM^ and Excel^TM^ were used to manage the data, with reflexive thematic analysis selected to analyse the data, by progressing through the six stages of thematic analysis as identified by Braun and Clarke [50]. This approach is an iterative process that involves moving back and forward between phases of analysis and returning to the raw data on occasions when necessary to improve understandings [50]. The familiarisation phase (Phase 1) involved CB reading and re-reading each transcript at length and relistening to the audio recordings to get a sense of the data set as a whole. Throughout this familiarisation process CB kept a diary of reflections. During phase 2 (coding), CB documented segments of the data by highlighting meaningful phrases and making notes, and then inductively assigned codes to sections of the data, similar to an approach used by Trainor and Bundon [63]. In the third phase (generating initial themes), CB clustered codes into candidate themes, where it became clear that initial codes shared a pattern of meaning across the transcripts. Candidate themes were iteratively reviewed and discussed with JM. Phase 4 (developing and reviewing themes) involved further discussion and rigorous debate of the candidate themes with CB, JM, GO’D and AK, with all authors challenging the assumptions they were making in interpreting the data, leading to the refinement and development of the themes [64]. This reflexive and collaborative process led to the generation of themes that were then deductively mapped (by CB) onto the domains of the TDF to act as a lens through which to view different influences on mothers’ PA support behaviours, and again iteratively reviewed and discussed with JM (See Table 2). During phase 5 (refining, defining, and naming themes) CB’s proposed thematic structure and inductive-deductive analysis was discussed and debated among the wider research team (GO’D and AK), resulting in further refinement of themes (See Table 2). Phase 6 (writing the report) involved continuous movement between stages five and six and the analysis presented in this manuscript was written. Pseudonyms have been used for identifying information to protect the anonymity of participants.

**Table 2:** The themes mapped to the Theoretical Domains Framework.

TDF domain (Definition^1^^)^	Illustrative quote	Theme development (Stage 4)	Final themes (Stage 5 & 6)
**Knowledge** (An awareness of the existence of something)	*You have to show them that it’s good for them and that you can enjoy it and have fun doing it*. (Sandra, 47, a mother of four)	Highlighting the benefits of being active	It’s what mothers do
	*Well*, *I think we’re lucky in the sense that there is a lot around for them*. *There’s a lot of clubs*, *numerous different football clubs*. *There’s a girls club*, *there’s loads of boys*. *There is a great variety of things*. *We are lucky in that sense that we’re not struggling to find things for them to do*. (Chloe, 41, a mother of three)	Knowing what activities are available (or not)	Our community shapes the support we provide
**Skills** (An ability or proficiency acquired through practice)	*“I’m not sporty*. *No*. *I mean*, *I’m here talking about how I think it’s so important for her to be on a team*. *I wouldn’t have been*, *I don’t think I’d be able to kick a ball out of my way now*. (Leah, 40, a mother of three)	Having the ability to be active	It’s what mothers do
**Social/Professional Role and Identity** (A coherent set of behaviours and displayed personal qualities of an individual in a social or work setting)	*“It’s what you do*. *It’s what mammy and daddy done and it’s what we done*, *it’s next*.. *and it’s generational*. *So you hand it down*.*”* (Amy, 42, a mother of three)	A mother’s role	It’s what mothers do
**Beliefs about Capabilities** (Acceptance of the truth, reality, or validity about an ability, talent, or facility that a person can put to constructive use)	*“What I find easy is I have a great interest in it*. *It’s not a burden for me to do the runs or go to the matches or go to the trainings and be on the side line*, *be in the freezing cold*. *I don’t find that a struggle because I’ve done it all my life so it’s just I’m not playing the game but I’m on side lines*. *Do you know what I mean*? (Amy, 42 a mother of four)	Believing you can provide support / be co-active	It’s what mothers do
**Beliefs about Consequences** (Acceptance of the truth, reality, or validity about outcomes of a behaviour in a given situation)	*“I think if we were lying around on the couch all day*, *she might follow*, *you know what I mean*? *I think*, *us moving around that and being active and that it would definitely influence her and encourage her*.*”* (Beth, 42, a mother three)	Our support encourages her to be active	It’s what mothers do
**Reinforcement** (Increasing the probability of a response by arranging a dependent relationship, or contingency, between the response and a given stimulus)	*“Well*, *I suppose the easy thing is that she wants to go*, *so it’s very easy then*. *There’s no row*, *there’s nothing like that*.. *For me then*, *when she goes to basketball*, *I go for a walk*. *When they go to Harriers*, *I go for a walk so it’s win-win*.*”* (Helen, 44, a mother of two)	Daughters reinforce their mothers’ behaviour	Negotiating the social complexities of providing support
**Goals** (Mental representations of outcomes or end states that an individual wants to achieve)	*“My goal is to get her into something that she can take into her teenage years and into her twenties…*.*But just to get her into something*, *I’d love her to have*.. *like that into her early 20s to have a sport that would you know benefit her in later life really is what I think*.*”* (Janis, 42, a mother of two)	Our support encourages her to be active	It’s what mothers do
**Memory, Attention and Decision Processes** (The ability to retain information, focus selectively on aspects of the environment and choose between two or more alternatives)	*“If there’s stuff available in the local town that’s obviously a help*. *We actually drive to Tullamore to bring them to basketball*. *I played basketball as a child*, *my sister is very into basketball*. *Basketball was something I was into*. *So we made that conscious decision to drive to Tullamore to bring them to basketball”* (Alma, 45, a mother of three)	Having to decide how to allocate time and resources	Our community shapes the support we provide
**Environmental Context and Resources** (Any circumstance of a person’s situation or environment that discourages or encourages the development of skills and abilities, independence, social competence, and adaptive behaviour)	“*Here yeah*, *so there’s definitely no sport options*. *There’s only two swings and there’s about 60 kids in the estate but that’s a sport itself*, *running to the swing before someone else…*. *So*, *we wouldn’t really go outside here in our own doorstep*.*”* (Joan, 35, a mother of one)	(Limited) access to facilities	Our community shapes the support we provide
**Social influences** (Those interpersonal processes that can cause individuals to change their thoughts, feelings, or behaviours)	*“I think a lot of it is about the parental support*, *because if the parental support isn’t there* … *And even between us*, *if I didn’t have Barry behind me*.. *If we weren’t talking about*, *‘We got to keep her in it*.*’ It’s a very slippery slope*.” (Elaine, 44, a mother of three)	The role of other members in the family	Negotiating the social complexities of providing support
	*Even for us*, *when we went to the community games final*, *all the parents who were there all brought down sandwiches*. *We all went back to the hall afterwards*. *Just so lovely to see*. *Even all the mammies*, *we all went for a drink afterwards* (Alma, 45 a mother of three)	Creating a community of mothers / families	Our community shapes the support we provide
**Emotion** (A complex reaction pattern, involving experiential, behavioural, and physiological elements, by which the individual attempts to deal with a personally significant matter or event)	*“I’m so proud she loves it* … *It’s great seeing her being so proud of herself*, *when she’s out there*. *It is great*.*”* (Chloe, 43, a mother of three)	It feels good to see my daughter active	It’s what mothers do
	*“We’re very rural so the school doesn’t have two and three pitches*. *The school has maybe one pitch for soccer and one pitch for Gaelic games and it’s the same school I went and boy did I fight in there over my rights to that pitch but like that is still there to a point*. *Girls get off*. *The pitch is booked for the boys*. *We’re having it now*, *get off*. *That boils my blood*. *And that maybe annoys me*. *But look*, *I think I have her taught enough to fight for her rights now*.? *If you’re on the pitch*, *you stay on the bloody pitch*.*”*(Sandra, 47, a mother of four)	Infrastructure challenges cause frustration	Our community shapes the support we provide

Note. ^1^Definitions based on Atkins et al., (2017) [49]

### 2.6 Validity

For this study, validity concerns the steps used to ensure the credibility and quality of the research [65]. Similarly, validity can refer to the accounts or conclusions reached with regard to particular methods, context and purposes [66]. In line with a critical realist perspective we applied recommendations provided by Ronkainen and Wiltshire [65] in establishing validity of our results. To establish empirical adequacy of the accounts, all interviews were recorded, transcribed verbatim and then re-read to check transcript accuracy. Moreover, to establish ontological plausibility, three authors acted as critical friends [67], allowing for reflectivity and clearer interpretation of results. This process included providing guidance and critical feedback of the first author’s assumptions at all stages of the analysis (i.e., coding, developing, and reviewing themes, writing the report). For example, through these discussions, our conceptualisation of maternal PA support was progressively refined as complex with multiple varied influences that shape mothers’ experiences.

We invited member reflections from participants by sending them preliminary results and inviting them to discuss these results with CB to explore if additional insight would be created through this dialogue [65]. However, no new themes were developed following these discussions. Finally, our research can be judged by its significance and worthiness, which contributes towards establishing validity via practical utility [65]. We view the outcomes of this study as an essential step towards understanding mothers’ experiences of providing support to keep their daughters physically active and will inform future intervention development and design. This is timely considering health systems globally continue to endure severe health and economic burden associated with non-communicable diseases, with an estimated cost to reach more than INT$520 billion for the period 2020 to 2030 if current levels of physical inactivity are not reduced [68].

## 3. Results

Three themes were developed to describe mothers’ experiences of supporting their daughters to be physically active and to understand their perceptions of the factors that might influence these experiences. These themes were situated at the individual, social and environmental levels, and were, ‘it’s what mothers do’, ‘our community shapes the support we provide’ and ‘navigating the social complexity of providing support’. In line with using the TDF as a theoretical lens to inform the study, the themes were also mapped to the relevant domains of the TDF (see Table 2). Together, this approach provides detailed knowledge of mothers’ experiences of supporting their daughters to be physically active, and the factors that might influence it which could be used to inform the development of future family-based PA interventions.

### 3.1 It’s what mothers do

Most mothers saw providing support for their daughters’ PA as an inherent part of their parental role. Their role in providing support was influenced by their own PA identity or lack thereof (e.g., ‘*I am someone who is active*’ or ‘*I’m not the sporty type’*) and by their confidence to be active. They also described the range of emotions they experienced in providing this support to their daughters. Mothers highlighted how their support could take various forms, for example, facilitating enrolment in activities, organising transport to activities, providing encouragement, spectating and even co-activity with their daughters. In discussing their daughters’ PA, mothers often conflated sport with PA, focusing on their daughter’s participation in organised sports rather than other forms of PA, with little reference to gendered activities. For most mothers, *“making friends”*, *“building confidence”* and establishing *“healthy habits”* represented what they would like to see as a result of providing support to their daughters. They recognised the importance of being actively involved in supporting their daughter, this was particularly relevant for daughters who were reluctant to partake in physical activities of various forms as explained by Alice, 43, a mother of two, *“If I wasn’t there to support her and bring her*, *she’d lose interest and it would stop*.*”* Several mothers discussed how providing support to their daughters was *“generational”* as it was passed on to them from their own mothers, thus, there was a deep rooted expectation to do it. Sue, 40, a mother of two remarked:

*“It’s just what you do*. *In fairness*, *it’s just done*. *It’s just what you do*. *It was always done*. *It was done for me*. *It would be done for her*. *Hopefully she will do it for hers if she ever decided*. *It’s just what you do*.*”*

An important contributing factor to this “passing on” of PA to their daughters was a mother’s own PA identity. This identity was typically constructed from their own experience of being active as a girl which then led to it being integrated (or not) into their adult life in some form. This was evident in how Helen, 44, a mother of two, described her positive experiences of PA as a girl and how that has led to a PA identity that is integrated within her sense of self as an adult:

*“Your own life experience really influences this*. *When I was in school*. *I was on teams and stuff*. *Now*, *I was maybe a sub*, *but I was able to do it*. *But I liked sport*. *I liked fun… And now*, *I just feel for myself that physical activity is just something you do*. *It’s not something you make time for whatever*. *It’s just that it has to be an integral part of just like the way you’d eat your breakfast*, *you’d just be going for a walk*.*”*

Furthermore, mothers’ robust PA identity also appeared to influence their daughter’s interest and participation in sport. This was illustrated by mothers highlighting how their passion and enjoyment of PA was often reflected by their daughters engaging in similar sports or activities. This was captured by Sandra, 47, a mother of four, who reflected how she “*loved camogie*. *So*, *I know that had an impact on the fact that the girls play*.*”* However, for other mothers with a less defined PA identity, they sometimes struggled to decipher how best to support their daughter to be active, balancing the recognition of its importance but not coercing them to do it. Oftentimes, this lack of identity was influenced by mothers’ own negative childhood or adolescent PA experiences, particularly regarding organised sport. This was illustrated by Beth, 42, a mother of three:

*“I’m not very sporty*. *I never did sports in school*. *Absolutely useless*. *End of the line was never picked*. *Always the last one to be picked*. *That’s probably why I have not ever really forced them*. *I know what it’s like to not be any good*, *so I’d never push them*.*”*

This lack of a defined PA identity in some mothers was associated with a lack of confidence in their own ability to be active, and this prevented them from co-participating in activities with their daughters or role modelling PA to their daughters. Ava, 44, a mother of three described how she was *“not very good at moving myself”*, and thus preferred not to engage in activities with or without her daughter. Indeed, many mothers referenced being *“sporty”* or *“not sporty”* which in turn influenced the type of support they provided to their daughters. However, for mothers with a defined PA identity, they reported it being *“easy”* to promote and support their daughters to be active. This confidence was sometimes enhanced by the perception that their daughters would listen to them as they had played a particular sport or did a particular activity themselves. Leah, 40 a mother of two described, *“When I would say things like you need to work on X*, *Y*, *Z*, *[*..*] She knows that what I’m saying is from experience and I think that definitely helps*.*”*

In describing their role in supporting their daughters to be active, mothers expressed a range of emotions they experienced from joy to frustration and fear. Several mothers noted pleasure and pride when watching their daughters participate in organised sport, as described by Sandra, 47, a mother of four, who reflected on the positive emotions associated with her experience of being on the side-line at her daughter’s matches:

*“Just watching them play*. *I just get goosebumps*. *I’m terrible*, *I get bored going to other matches*. *I just love*, *love*, *love watching them out in the open field*, *running around*, *striking a ball*. *I just love it*. *It’s not my life*, *but I do get so much joy and satisfaction from seeing them play*. *I just love it*.*”*

In contrast, some mothers expressed frustration at their daughters’ indecision towards participating in activities, for example joining a new sports club or taking part in after-school activities. Liz, 43, a mother of two, described this frustration, sharing how her daughter alternates between enthusiasm and hesitancy towards taking part, *“So I said “Right Lucy*, *don’t do it*. *Fine*.*” But then it’s like “Oh*, *I will do it*.*” It’s so frustrating*, *but she’s decided to do it*. *[*..*] So she will go through swings and roundabouts*.*”* In these situations where daughters were reluctant to take part in activities, mothers also expressed fear of *“pushing too much”* or being afraid of *“putting her off altogether”* and therefore often stopped providing support. Janis, 42, a mother of two, detailed, *“There’s a certain amount that you can push too*, *and then I feel like I have to step back*, *I don’t want to upset her altogether you know and traumatise her*.*”*

### 3.2 Our community shapes the support we provide

Mothers recognised that the type and amount of support they provided was also impacted by the community setting in which they lived and in particular by the infrastructure within their community. This manifested itself in mothers expressing considerable reluctance to promote outdoor play or active travel to their daughters due to a fear for their daughters’ safety. However, there was divergence in mothers’ experiences of this based on living in an urban or rural area. Anti-social behaviour was the common concern for mothers in urban areas as highlighted by Joan, 35, a mother of one:

*“Where I live*, *unfortunately*, *there’s no*.. *actually just a very small playground*, *for a whole lot of kids*. *She wouldn’t really like going out and playing or getting involved as much as I like because there would be anti-social behaviour sometimes*.*”*

Whereas mothers living in rural areas described road safety and lighting as the major concerns. Many of these mothers expressed fear of the roads as a reason for prohibiting their daughters to walk or cycle to school or to their organised activities. Tanya, 44, a mother of two, described:

*“We live in the country*, *so we do have access to nice walks*. *But then the other side is we don’t have cycle tracks*. *The road is very busy*, *for a cycle it can be quite dangerous*. *You couldn’t cycle to school or do anything like that because it just would be too dangerous*. *And then if they’re going for an actual specific activity or an organised activity*, *they have to be driven*.*”*

Local sports clubs were considered by many mothers as an important facilitator in providing support for their daughters’ PA. These clubs were typically perceived to be at the centre of the community, particularly in rural areas, and therefore not only afforded their daughters the opportunity to take part in PA but also gave mothers the chance to engage with other families in the community. The social relationships they cultivated with these other mothers, tended to reinforce their motivation to continue providing support to daughters. For example, Alma, 45, a mother of three shared, *“I would go to all the football matches*. *You’re getting to know a whole different cohort of people through that*. *And it’s lovely*.*”* Similarly, Tanya, 44, a mother of two, described her experience of volunteering at her local sport club, *“And it’s social*, *you get to chat to other mothers*. *You get to know people you wouldn’t normally see much of*.*”*

While sports clubs were viewed as an important facilitator by mothers, they also voiced concerns over certain factors within these clubs. For example, instances where resources at a club were limited (e.g., access to pitches, the availability and quality of coaches), mothers felt priority was often given to boys, and expressed frustration with the *“traditionalists”* who were still involved in the clubs. In some instances, these gender issues led to mothers taking their daughters out of clubs. Emer, 44, a mother of two, living in a small rural community in which the sport club acted as a social hub for the town remarked:

*“I do find challenges*. *I don’t like the boy-girl divide […] that is still there to a point*. *Girls get off*. *The pitch is booked for the boys*. *We’re having it now*, *get off*. *That boils my blood*. *And that maybe annoys me*. *I see it all the time*. *There’s less spectators at the girls’ matches*. *That’s just the truth of it*. *And I think what happens at the girls’ matches is it’s the immediate family and maybe a few*, *but the town don’t come out to watch them*.*”*

Some mothers viewed the local school as an important community resource to assist with supporting their daughter’s activity. This was especially prevalent with mothers who felt they were unable to support their daughters’ involvement in sports clubs, for reasons such as lack of choice, time constraints or financial concerns. These mothers believed having organised activity during and / or after school might encourage their daughters to become more involved in PA and offer a more realistic way in which to encourage their daughters to be active. This was particularly evident for mothers who were in full time employment, separated from their husbands or single parent families. Leah, 40, a mother of three who works full-time reflected, *“the practical side of it (scheduling activities) can be difficult*. *I suppose the other side*, *it’s making time*. *It is just making time*. *But there is that logical side to it*, *it can be hard*. *I’d be lying if I said it wasn’t*.*”*

### 3.3 Navigating the social complexity of providing support

Mothers acknowledged that they did not provide support for their daughters’ PA in a social vacuum, and how they and their daughters engaged with other people influenced the amount and type of support they could provide. Mothers described the assistance provided by partners as playing an important role in influencing how they supported their daughters’ activities. Many fathers were involved in the practicalities of providing PA support within the family as described by Rose, 47, a mother of two:

*“And then*, *if I was a single parent*, *I couldn’t do what I did*. *So*, *I have my partner as well*, *my husband*. *So he brings one child one place*, *and I can split and bring the other child the other place*, *because there’s two things going on*, *some nights for one child and another*.*”*

Similarly wider family members were also recognised by mothers for the contribution they made towards supporting with the practicalities of providing support as shared by Alice, 43, a mother of two, who works full time, *“My in-laws take her the days that I’m not here*. *If I asked them to bring her to the moon they would*, *to be fair to them*.*”*

Oftentimes, a mother’s role in supporting their daughter was reduced due to a partner’s involvement in their daughter’s activity. Mothers who identified their partners as *“sporty”* or *“outdoorsy”* were often inclined not to take part in co-activities with their daughters as they felt their daughter would have a more enjoyable experience with their father. This was particularly evident in mothers who perceived themselves as not being an *“active person”*. As an example, Helen, 44, a mother of two, described how she felt her husband was more suitable to be active with their daughter:

*“Now*, *he’s very good*, *but he’s very outdoorsy*. *He likes to go for walks and stuff*, *but he brings them for cycles…*.. *I make no secret to the fact I hate cycling*, *so sometimes the children and George do it*, *but I just hate every bit of it*. *Hate it*.*”*

Mothers also highlighted how interactions with their daughters reinforced the support they provided to them. For many mothers, this was driven by their daughter’s inherent interest or not in being active. Helen explained how her perception of her daughter’s preferences, *“she will gravitate towards being inside*, *sitting at the table*, *drawing*, *that sort of thing”* meant she tended to promote those types of activities over PA to her daughter. This was particularly challenging when there were multiple children in the family with different preferences sometimes leading to conflict and ultimately, physical inactivity due to a lack of consensus. This was illustrated by Pam, 41, a mother of twin daughters who had different activity preferences:

*“Because I try my best*, *but then it ends up in fights and ruins the day*.

*Things like*, *oh*, *it’s a nice day outside*. *Come*, *let’s go to somewhere*. *And then one is agreeing*, *the other one is disagreeing*. *Really much disagreeing*. *And then it’s fighting*. *And then I’m like*, *I’m not going anywhere*.*”*

Equally, mothers who believed their daughters *“had a natural interest”* in being active felt it was easy to support them, and this led to increased support by these mothers, and increased activity by their daughters. Mothers also described receiving positive feedback from their daughters and how this provided reinforcement to continue providing support as illustrated by Sandra, 47, a mother of four:

*“She said to me probably two weeks ago*, *mammy*, *thanks so much for teaching me how to play camogie (stick-and-ball team sport) because I don’t know what I’d do without it* … *it did mean a lot to hear her say that to me*, *to be honest*, *I was like oh God*, *you’re breaking me heart here*. *It was lovely at the same time for her to feel that she really does enjoy it because there’s nothing worse than a child being forced to play something and they don’t enjoy it*.*”*

Alongside the role of partners, and mothers’ own interaction with their daughters, all mothers, without exception, recognised the influential role siblings and friends had on their daughters’ enthusiasm and desire to be active, and how that could impact the support they provided. Mothers described occasions where the absence of their daughter’s friends impacted their daughter’s willingness to participate in activities regardless of the amount or type of support provided by them. Mothers felt this encouragement from friends played a vital role in helping them to introduce their daughters to new sports or activities, as explained by Leah, 40, a mother of two:

*“And then some of Grace’s friends were involved in the club and she said*, *I’d like to give it a go*, *mam*. *And I said that would be brilliant*. *Yeah*, *absolutely*. *Let’s give it a go*, *see if you like it*. *And that’s kind of how it all came about*.*”*

Importantly, some mothers also highlighted the negative influence peers could have on their daughter’s activity, particularly boys, where their daughters were intimidated or upset by comments that boys made. In many cases, this led their daughters to disengage from an organised sport or to stop engaging in certain activities in playgrounds or parks. Helen, 44, a mother of two, described:

*“And the boys think it’s okay to say you’re slow or you’re not fast enough or whatever they want to say*. *Boys say things*, *and that’s allowed*. *And all of a sudden*, *the girl says*, *‘I’m taking myself out of this*.*’ And that’s where you lose the girl because they actually have to not just fight against other girls to keep their space*, *they have to fight against the boys too*.*”*

### 3.4 Mapping themes to the Theoretical Domains Framework

**Fig 1:**
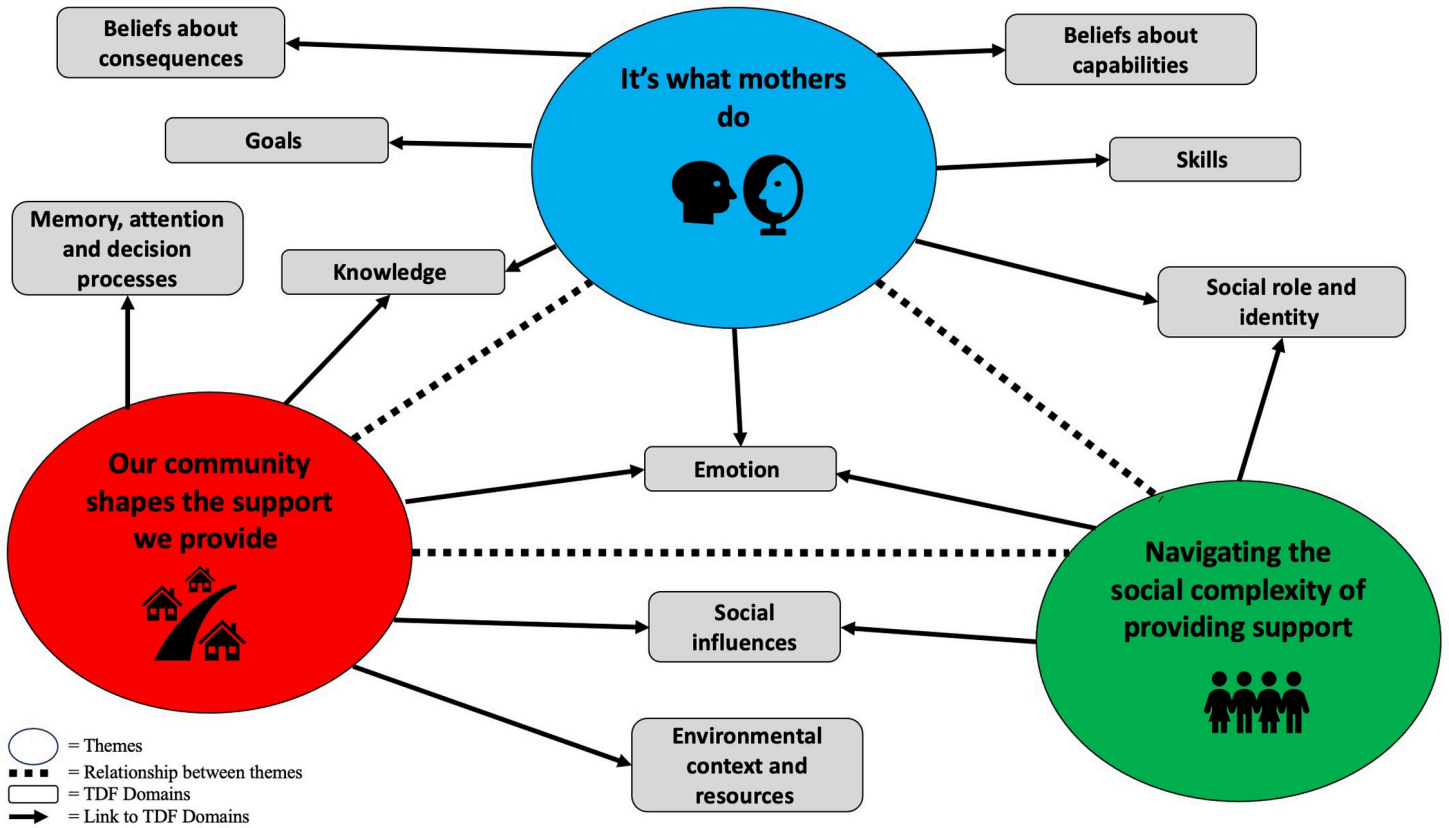
Thematic map demonstrating interactions between identified domains of the Theoretical Domains Framework and themes.

The themes were mapped to the TDF domains where 11 of the 14 domains were considered important (illustrated in Table 2). A thematic map is presented in Fig 1 showing the complexity of factors that influence mothers’ supportive behaviours with certain TDF domains represented across (see Fig 1).

The domain of knowledge was present through mothers’ understanding of different types of supportive behaviours, and their awareness (or lack of awareness) of different activities in their local environment for their daughters to engage in. Mothers’ beliefs about their capabilities and skills to support their daughters was evident primarily through their beliefs about their own ability to be physically active and to engage in co-activity with their daughters. The domain of social role and identity was illustrated by mothers’ perceptions of their support being part of the good mother ideal, and the expectations they placed upon themselves. Mothers also discussed the outcomes they would like to achieve by supporting their daughters to be active, reflecting the TDF domains of goals, and beliefs about consequences. These outcomes tended to focus on their daughters’ personal and social development rather than achievement outcomes. A wide range of emotions experienced by mothers in trying to support their daughters to be active mapped to the TDF domain of emotion, from the joy they experienced watching their daughter taking part in sport, to the frustration they sometimes felt, e.g., with sports club officials who prioritised boy’s participation over girls. Reinforcement was present in how mothers were motivated to continue supporting their daughters to be active by the positive feedback they received from their daughters. The domain of environmental context and resources was highlighted in how mothers’ local environment assisted or inhibited their ability to support their daughters to be active, for example, access to safe playgrounds, the proximity of sports clubs or the availability of after school activities. Closely aligned to this was the domain of memory, attention and decision processes which was evident through mothers’ decision making as to how they could support their daughters to be active, based on the time, location and / or financial constraints they faced. Finally, the TDF domain of social influences was present in various forms. These included, mothers negotiating their partner’s role in supporting their daughter’s PA, the influence of the wider family network, the connection they built with other mothers through their daughters’ activities, and how their daughters’ peers could amplify or thwart their attempt to support their daughters to be active.

## 4. Discussion

The aim of this study was to explore, mothers’ experiences of supporting their daughters to be physically active and, their perceptions of the factors that might influence these experiences. These results extend our knowledge of the complex and interrelated factors that shape how mothers support their daughter’s PA, with themes extending across individual, social, and environmental levels. Themes reflected how mothers saw supporting their daughter to be active as an inherent part of their parental role which was influenced by their own PA identity. Circumstances relating to the mothers’ environment and the infrastructure within their environment influenced the type of support they provided. Furthermore, a range of social interactions were also instrumental in facilitating or inhibiting how mothers support their daughters to be active. The themes were also mapped to the TDF domains, presenting an in-depth behavioural diagnosis.

Most mothers recognised the importance of supporting their daughter to be active and viewed it as an inherent part of their role. This aligns with much of the research exploring PA engagement by mothers, and the ethic of care concept whereby mothers put the needs of their children first [33]. For many, this belief was underpinned by their identity as an active or sporty person which was influenced by their own experiences as a child or adolescent [36]. Mothers who reported a more defined PA identity tended to also be more confident as to how they could support their daughters to be active. Interestingly, mothers did not discuss traditional gender roles as being a barrier to providing PA support to their daughters. Previous research has highlighted the pressures women can feel to fulfil traditional roles such as “mother”, “partner”, and “homemaker”, and this can reduce engagement in other activities such as PA [37]. In the present study, most mothers were in employment, had a partner, and multiple children, however, these other roles did not appear to lessen the PA support they gave to their daughters. This may be due to mothers perceiving PA support as part of the good mother ideal [33] or PA support extending beyond role modelling or co-participation of PA to other forms of support such as encouragement and logistical support which could be less time consuming, and thus less demanding on mothers’ time. These wider findings on PA identity are in line with research by Rhodes, Berry et al, (2019) [69] who demonstrated that parental identity towards providing PA support was critical for implementing support, therefore, strengthening maternal identity regarding activity may increase the likelihood of a mother’s enactment of PA support. This is likely to be particularly important for mothers who don’t identify as an active person. While, preliminary research among university students suggests targeting identity related concepts is feasible [70], there is a paucity of published identity-based PA interventions, thus future research in this domain is warranted. Mothers’ supportive behaviour was also influenced by their beliefs about their confidence to provide support. Oftentimes, mothers displayed strong intentions to support their daughter, however difficulty enacting these intentions arose due to a lack of confidence to be active themselves. Recent research has highlighted that building a parent’s competence to be active enhances the values they hold towards PA which can ultimately enhance a child’s values towards PA [71].

Social support from mothers’ family members and their wider social network were important factors that shaped how a mother supports her daughter’s PA. From a family systems perspective [44, 72], a family self-regulates through feedback loops within different interdependent subsystems (e.g., mother-daughter; father-daughter, mother-daughter-father), where change in one sub-system may influence change for the entire family [73]. This complexity was evident through the role, partners or grandparents often had in supporting their (grand)daughters to be active, and how these roles can enhance or supplant a mother’s attempt to support their daughter to be active [43, 74]. For example, where mothers had low levels of confidence to be active with their daughter, partners (typically fathers) engaged in these activities with their daughters instead. While research suggests fathers do engage in co-participation activities with their children, this tends to be with their sons rather than with their daughters [74]. However, qualitative research has highlighted the positive emotions experienced by daughters when they do co-participate in PA with their fathers [75]. Our research also found mother-daughter interactions were represented by both negative (e.g., arguments with daughters about engaging in PA) and positive experiences (e.g., co-participating). This is noteworthy as this feedback from daughters reinforced mothers’ beliefs and intentions towards the type of support they provided, for example, mothers who met resistance when organising activities, found not having their daughter on board more challenging, if not impossible to support or led to mothers disengaging from the supporting role due to the conflict created. Thus, education interventions focusing on how mothers can communicate positively with their daughter could in turn improve parenting practices for PA such as, encouragement, guided choice and co-participation [76]. Our results also shed light on how mothers’ enjoyment of supporting their daughter to be active was enhanced by the social relationships they developed with other mothers which then increased their motivation to continue providing support. All mothers in the study described the importance of their daughters having friends at their activities. There is a large body of research highlighting the importance of peer support for girls’ PA [14, 77, 78] therefore, future research should include peer support to facilitate mothers’ support behaviours and increase daughters’ enthusiasm towards being active.

Our results also acknowledge how environmental factors such as local community infrastructure and contextual factors can facilitate or inhibit mothers’ promotion of PA to their daughters. Mothers described how anti-social behaviour in urban areas and road safety in rural areas, were barriers to promoting outdoor play and active travel respectively. These results are in line with previous research citing neighbourhood safety concerns as one of the main barriers for parental support of children’s outdoor play [79] and active travel [80]. A recent review suggesting improvements to infrastructure (e.g., bike paths, street lighting and playgrounds) as a method for promoting PA in children and youth [81], could create the physical environment that fosters mothers’ support for daughters’ activity. Accessibility to local sports clubs and organised school based activities was emphasised by many mothers as facilitating their daughters’ involvement in PA. This highlights the importance of accessibility and convenience of available recreation and sports programs via community sports clubs and schools to enable children’s PA and promote parents’ engagement in support behaviours [20, 82]. When it came to organised activities such as in local sports clubs, mothers had to consider the expense of participation, alongside access to a car or the cost of public transportation when deciding whether to enrol their daughter. While this is not a new finding, it illustrates again the difficulties for mothers regarding their daughter’s participation in organised sport [83, 84], particularly those from lower income families [85].

### 4.1 Practical implications

The themes identified in this study provide insight into the complex interplay of individual, social and environmental levels factors influencing maternal support for their daughters’ PA. By mapping these themes to the TDF, it allows us to suggest how these themes could be targeted in future interventions. The first theme was underpinned by mothers’ own PA identity (TDF: social and professional role), their confidence to provide this PA support (TDF: beliefs about capabilities) and the emotions they experienced in giving this support (TDF: emotion). To strengthen mothers’ PA identity, the use of behaviour change techniques such as mental imagery have shown promise in strengthening role related identity with respect to PA in women [86]. Furthermore, increasing mothers’ confidence to engage in supportive behaviours appears important as does promoting positive affect. There is evidence to suggest that techniques such as providing information on the consequences of the behaviour, providing instruction on how to engage in the behaviour, and reinforcing the effort towards the behaviour, can facilitate the development of a person’s confidence to be active [87]. Furthermore, a recent review has highlighted how techniques such as social comparison might promote positive affect for PA by eliciting comparisons with others [88], in the context of the current study, perhaps by mothers comparing their PA alongside that of their daughters. There were considerable social complexities evident in how mothers engaged in supportive behaviours (TDF: social influences) ranging from their engagement with their daughters regarding PA, to how they utilised their social network to cultivate positive relationships with other mothers. There are several behaviour change techniques that could influence some of these interpersonal interactions. These might include encouraging positive reinforcement (e.g., expressing gratitude) from other family members when a mother provides support to their daughter; developing mothers’ communication skills such as active listening to enable them to engage positively with their daughters about PA [89] and social comparison, promoting mothers to compare experiences and behaviours with those of other mothers to gain insights into how to improve their support behaviours, and build a support network beyond the family [90]. At the environmental level, the communities’ mothers lived in influenced the support they could provide their daughters. There is a growing body of evidence to indicate that increasing safe opportunities for children to engage in play-based activities out of school may be a worthwhile strategy to pursue [91]. An innovative approach might be the introduction of “play streets”. These are the temporary closure of streets for a specified period of time, typically on a regular basis, which enables the creation of safe, publicly accessible space for children, and their families to engage in PA. There is some evidence that this approach can increase overall PA in children [92]. Alongside this, increasing community investment strategies to make sport or other organised activities more accessible and affordable would be an effective pathway for developing and improving mothers’ opportunities to support their daughter to be active [43]. For example, a free community wide PA pass programme has shown promise in promoting increased PA and organised sport access to pre-teen girls, particularly for girls from lower income families and when located with two kilometres of the girls’ homes [93]. Intervening at each of these levels is important in promoting PA. However, each of these levels is likely to connect with each other, and thus, there is a need for future interventions to consider how these components might interact to facilitate maternal support of PA [17]. Indeed, there is emerging evidence to support interventions involving both the family and the school environment to increase PA behaviour, particularly when there is participation and pursuit of relevant goals by both family members and children [94].

### 4.2 Strengths and limitations

This study adds to the limited number of qualitative studies that have explored mothers’ supportive behaviours for PA, and a provides a rich exploration, enabling an increasing understanding of why, where and with who, mothers provide support to their daughters. A detailed behavioural diagnosis using the TDF was conducted with a number of relevant theoretical domains identified. These domains could be used to determine specific theories (e.g., self-determination theory, [95], or family systems theory [72] to underpin future family-based support interventions. However, it must be noted that use of the TDF as part of the analysis could restrict results [96]. To address this and in line with recent recommendations, the reflexive thematic analysis was both inductive and deductive with three themes created which were then mapped to the TDF to develop a detailed interpretation of the data [62]. There is also a need to consider the views of a range of stakeholders, for example, daughters’ own experiences of receiving maternal support to be active, other family members and the role of community organisations. Indeed, future interventions should incorporate the views of stakeholders into the design process to ensure feasibility, and acceptability of the intervention to the targeted cohort [97, 98]. This is one of the first studies that has targeted mothers’ lived experiences of providing support for their daughter’s PA, however it must be acknowledged that the majority of mothers were White Irish, had some level of higher education and were married or in other partnerships. Additional research should consider engaging with more diverse populations, for example, mothers from low socioeconomic position, single parent families, LGBTQIA+, ethnic and religious backgrounds and people with disabilities, to gain insight into their experiences. Finally, while the lead author defined the term PA to mothers at the start of each interview, many mothers focused on how they supported their daughter’s engagement or not in organised sport rather than other forms of PA unless prompted by the lead author. Future studies should consider how to ensure the continuum of physical activities is considered by participants.

## 5. Conclusion

To conclude, the research adds to a small number of studies exploring mothers’ experiences of supporting their daughters to be physically active. Three themes were identified representing individual, social and environmental factors that influence these experiences. These results extend our knowledge of the complex and interrelated factors that shape how, where and why mothers provide support for their daughters’ PA. Additionally, the TDF was used as a theoretical lens through which to examine these complex factors, and how they could be used to inform future theory-based interventions to promote PA through aligning with potential theories and / or behaviour change techniques. Overall, the present study emphasises the importance for future researchers and intervention designers to consider individual, social and environmental factors when exploring how parents can support their children to be physical active.

## Supporting information

S1 File SRQR checklist. (DOCX)

S2 File Interview schedule. (DOCX)

S1 Checklist Human participants research checklist. (DOCX)
