# Supplementary material for: “It’s what mothers do.” A qualitative exploration of mothers’ experiences of supporting their daughter to be physically active
Source: PLoS One. 2024 Apr 1;19(4):e0299260. doi: 10.1371/journal.pone.0299260 (PMC10984520; doi:10.1371/journal.pone.0299260)
Supplement: S2 File — (DOCX) [file pone.0299260.s002.docx]

**Supplementary material 1: Semi- structured interview guide**

To respect conversation flow and to facilitate a comprehensive understanding of the factors related to maternal PA support, broad open-ended questions based on the theoretical domains framework were used in line with the recommendations by (McGowan et al., 2020).

**Section A:**

**Introduction**

Welcome and thank participant for taking part.

Introduce the study to participant and reasons for taking part. (i.e., researching ways to promote physical activity in 10-12 year old girls and looking for mothers’ experiences of supporting girls to be physically active to help with the research)

Reassure mothers that there are no right or wrong answers and get verbal consent to continue and record the conversation.

Questions below are numbered with bullet points as prompts

In circumstances where mothers were not supporting their daughter to be active we altered the question appropriately.

**Level of physical activity for mothers and daughters**

**DEFINE PHYSICAL ACTIVTY FIRST:** *For daughter, participation in organised sport, active travel e.g. Walk or cycle to school. Activities as a family, walking, swimming, bike rides., running around outside playing.*

1. How physically active is your daughter?

- What does she do?
- How do you think she feels when she’s active?

2. What do you think the benefits of being physically active are?

- For your daughter?
- For you?

3. What’s your sense of how much activity she needs to do?

4. How do you see yourself with respect to physical activity?

- For example, I am an active person/ I don’t like to be active/ I enjoy taking part in sports/ I used to like sports
- What do you do?

**Section B:**

**How mothers support their daughters to be active, and the factors influencing support.**

5. What is your role in your daughter’s activity?

- How do you view your role?
- What do you find difficult or easy?
- Wider role of family?

6. How does supporting your daughter make you feel? OR How would it make you feel?

7. How confident do you feel to support your daughter to be active?

8. What are the outcomes of supporting your daughter to be active? OR What might be the outcomes of supporting your daughter to be active?

9. What concerns you about supporting your daughter to be active?

- Now
- In the future

10. How do you / your daughter decide what activities or sport your daughter takes part in? OR how would you / your daughter decide what activities or sport your daughter takes part in

- Other family members / time constraints, finances, homework, family commitments

11. How does your local environment (where you live) influence how you support your daughter to be active? OR how you could support your daughter?

- Facilities / safety / schools / sports clubs / green spaces

12. What other factors might help / hinder how you support daughter to be active?

- Other family members / time constraints, finances, homework, family commitments

13. What other influences are there on your daughter’s level of activity?

- Friends, father, siblings, coaches, famous athletes / school / sport clubs

**Section C: Summary**

15. Is there anything we have not discussed about your experience of supporting your daughter to be active that you think is important?

16.Have you any suggestions or anything else that you would like to add?

Finish by informing mothers that interviewer will be in touch in due course, to show the findings and discuss with them any additional insight they might have.

Thank you very much for your time.

McGowan, L. J., Powell, R., & French, D. P. (2020). How can use of the Theoretical Domains Framework be optimized in qualitative research? A rapid systematic review. *British Journal of Health Psychology, 25*(3), 677-694.
